# Construction of Photoresponsive 3D Structures Based on Triphenylethylene Photochromic Building Blocks

**DOI:** 10.34133/2022/9834140

**Published:** 2022-09-02

**Authors:** Xiayu Zhang, Fukang Liu, Beibei Du, Rongjuan Huang, Simin Zhang, Yunfei He, Hailan Wang, Jingjing Cui, Biao Zhang, Tao Yu, Wei Huang

**Affiliations:** ^1^Frontiers Science Center for Flexible Electronics, Shaanxi Institute of Flexible Electronics & Shaanxi Institute of Biomedical Materials and Engineering, Northwestern Polytechnical University, 127 West Youyi Road, Xi'an 710072, China; ^2^Key Laboratory of Flexible Electronics & Institute of Advanced Materials, Nanjing Tech University, 30 South Puzhu Road, Nanjing 211816, China; ^3^State Key Laboratory of Organic Electronics and Information Displays & Institute of Advanced Materials (IAM), Nanjing University of Posts & Telecommunications, 9 Wenyuan Road, Nanjing 210023, China

## Abstract

Photoresponsive materials have been widely used in sensing, bioimaging, molecular switches, information storage, and encryption nowadays. Although a large amount of photoresponsive materials have been reported, the construction of these smart materials into precisely prescribed complex 3D geometries is rarely studied. Here we designed a novel photoresponsive material methyl methacrylate containing triphenylethylene (TrPEF_2_-MA) that can be directly used for digital light processing (DLP) 3D printing. Based on TrPEF_2_-MA, a series of photoresponsive 3D structures with reversible color switching under ultraviolet/visible light irradiations were fabricated. These complex photoresponsive 3D structures show high resolutions (50 *μ*m), excellent repeatability (25 cycles without fatigue), and tunable saturate color degrees. Multicomponent DLP 3D printing processes were also carried out to demonstrate their great properties in information hiding and information-carrying properties. This design strategy for constructing photoresponsive 3D structures is attractive in the area of adaptive camouflage, information hiding, information storage, and flexible electronics.

## 1. Introduction

Photoresponsive materials are a type of smart materials that show obvious physical or chemical properties changes under light stimulus. Due to the advantages of light, such as nondirect contact, easy access, high-precision, wavelength-dependence, and no by-products [1–7], a growing number of light-responsive materials are developed and involved in the fields of anticounterfeiting detection [8, 9], information storage [10, 11], chemosensing [12, 13], bioimaging [14, 15], smart adsorbents [16, 17], and molecular machine [18–20]. As a typical photoresponsive phenomenon, photochromism displays reversible chemical reactions and obvious color changes upon the light irradiation (with a certain wavelength). A series of organic photochromic systems, such as dithienylethene [21–24], azobenzene [25–28], spiropyran [29–31], fulgides [32, 33], and their derivatives, have been designed and profoundly investigated. However, the applications of such molecular photochromic compounds were mainly demonstrated by doping or grating in polymer matrices to construct simple 2D films or three-dimensional (3D) shapes [34–37]. With the booming development of photochromic materials, the construction of stable macromolecular photochromic materials in precisely prescribed complex 3D geometries becomes an appealing topic.

3D printing, a technology that refers to the additive manufacturing process of building 3D solid objects from digital models, shows the capability of customizing complex 3D structures [38–42]. Along with recently rapid developments in 3D printing, a few attempts have been made to fabricate photoresponsive structures with complex geometries by 3D printing, which showed enormous potential in the applications of adaptive camouflage, information hiding, information storage, soft robotics, and flexible electronics [43–47]. For instance, the commercial 3D-printed powders (*i.e.,* nylon 12) mixed with WO_3_ uncoated nanoparticles were used to create photoresponsive 3D structures by sintering-based 3D printing [48]. Hybrid organic-inorganic polyoxometalates were stabilized and solubilized by polymeric ionic liquid matrices, which could be further processed by 3D printing into photochromic 3D geometries [49]. Photoswitchable donor-acceptor Stenhouse adducts (DASAs) were postmodified on a two-photon laser processing complex microstructure [50]. However, due to the employed doping or postmodifying methods, the photoresponsive 3D structures suffered from unquantified and nonhomogeneous functional partials and poor solvent or thermal resistances. In addition, multicomponent 3D printing processes are hard to conduct with postmodify photochromic materials. Recently, triphenylethylene derivatives, which own admirable repeatability and simple chemical structures, are considered as promising photochromic materials [51–54]. Moreover, the easily modified chemical structure makes them promising candidates for preparing ultraviolet (UV) curable photochromic resins that are suitable for UV-based 3D printing technologies to directly establish macromolecular cross-linking network, such as digital light processing (DLP)-based 3D printing, a technology that is recognized as one of the most efficient 3D printing method to realize high-accuracy 3D structures with high speed [55–57].

**Figure 1:**
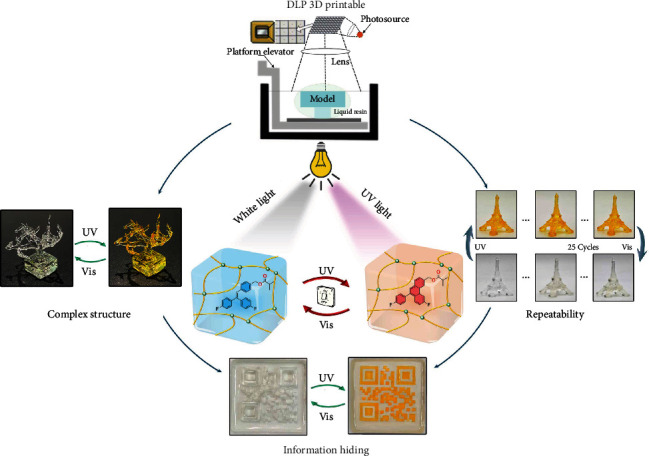
Comprehensive feature of 3D-printable photoresponsive materials.

**Figure 2:**
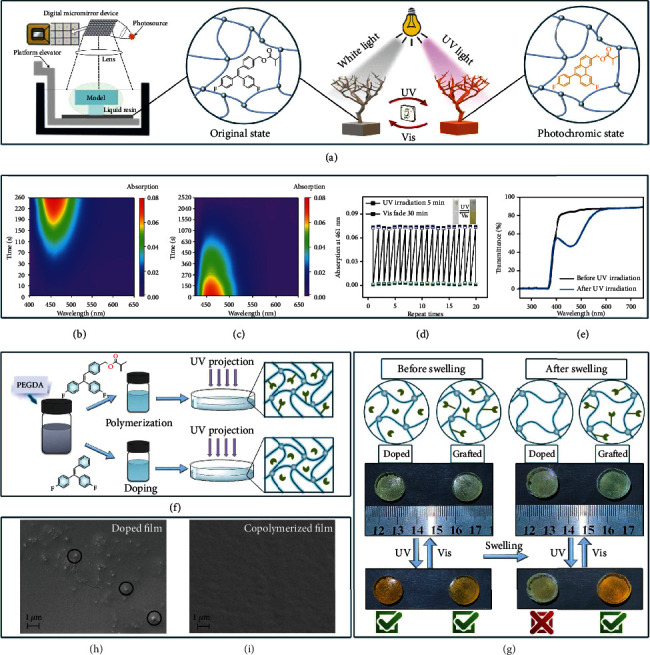
(a) Illustration of the photoresponsive material using the DLP 3D printing technology. (b) Time-dependent UV-Vis absorption spectra of TrPEF_2_-MA in degassed THF solution (1.0 × 10^−1^ M) during the irradiation process. (c) Time-dependent UV-Vis absorption spectra of TrPEF_2_-MA in degassed THF solution (1.0 × 10^−1^ M) during the bleaching process. (d) Recycling of the photochromic processes for TrPEF_2_-MA in solution state as a function of exposure to UV light (365 nm) and visible light for 5 minutes and 30 minutes, respectively. (e) The transmittance of the TrPEF_2_-MA polymer films with the thickness of *ca.* 2 mm before and after UV irradiation. (f) Different preparation processes of TrPEF_2_-MA containing doped and copolymerized films. (g) Comparison of photochromic properties between doped and grafted materials before and after swelling. SEM images of TrPEF_2_-MA containing doped (h) and copolymerized (i) films.

Herein, we demonstrate a simple and novel photoresponsive material, methyl methacrylate containing triphenylethylene (TrPEF_2_-MA), which can be directly used for DLP 3D printing for the first time (Figure 1). The high-resolution 3D objects show great solvent/thermal resistance and precisely controllable photoresponsive properties. TrPEF_2_-MA with a simple chemical structure exhibits controllable photochromism and fast response. More importantly, the MA moiety in TrPEF_2_-MA endows the molecule with UV curable ability, which could be directly copolymerized during the DLP 3D printing process (Figure 2(a)). By adding molecule TrPEF_2_-MA and photoinitiator into poly(ethylene glycol) diacrylate (PEGDA), liquid resins for high-resolution DLP 3D printing were successfully prepared. A series of highly complex and precisive 3D structures with high resolution up to 50 *μ*m were fabricated. Under the alternating UV and visible light irradiation, these printed stable macromolecular-based 3D structures featured reversible color switching between transparent and yellow (absorption at *ca.* 470 nm) with good repeatability. Besides, the saturate color degree of the photoresponsive 3D structures could be precisely controlled by adjusting the composition ratio of TrPEF_2_-MA in the resin. To further demonstrate the potential applications of the directly DLP 3D-printable photoresponsive material TrPEF_2_-MA, multicomponent 3D printing processes were carried out for the construction of the information (QR codes) hiding 3D structures, multicolor photoresponsive 3D structures, and information-carrying 3D structures. This study provides a novel strategy to directly design DLP 3D-printable photoresponsive material and reveals the potential applications of the areas of adaptive camouflage, information hiding, information storage, and flexible electronics.

## 2. Results and Discussion

### 2.1. Synthesis and Photochromic Properties of TrPEF_2_-MA

The synthetic details of 4-(2,2-bis(4-fluorophenyl)vinyl) benzyl methacrylate (TrPEF_2_-MA) are described in the Supporting Information. All compounds were characterized with ^1^H NMR spectroscopy and high-resolution mass spectrometry. The 3D-printable photochromic material TrPEF_2_-MA was achieved with decent synthetic yields of 37.3% through a simple three-step synthetic route as shown in Scheme S1 in Supporting Information. The low-cost starting materials, simple synthetic route, and high synthetic yields provided the possibility of large-scale preparation of TrPEF_2_-MA.

Upon UV excitation (365 nm), noticeable photochromism of TrPEF_2_-MA was observed both in solution and thin film. The color changes of TrPEF_2_-MA was ascribed to the reversible ring-closure reaction of triphenylethylene group [51], as illustrated in Figure 2(a), according to previous literatures with similar structures. In THF solution, a low-energy absorption band with the maximum at 461 nm was observed, which gradually increased with the increasing irradiation time. After irradiation for *ca.* 260 seconds, enhancement of the absorption band stopped (Figure 2(b)). With consequent visible light irradiation, the color of TrPEF_2_-MA solution gradually faded to its original color in 2520 seconds (Figure 2(c)). Moreover, thermal-back reaction (ring-opening reaction in dark) of TrPEF_2_-MA in THF (1.0 × 10^−1^ mol/L) was also achieved according to the fitting curve of the absorbance band at 461 nm in the time-dependent absorption studies. The thermal-back reaction is relatively slow, and the half-life in THF solution is 597.5 seconds in dark at 303 K (Figure S6). In order to provide evidence for photochromic mechanism and identify the ring-closure structure of TrPEF_2_-MA. By exposing TrPEF_2_-MA under UV irradiation in oxygen atmosphere for 12 hours, dehydrogenated product TrPEF_2_-MA(O) was obtained. As shown in Scheme S2, it can be concluded from the chemical structure of TrPEF_2_-MA(O) that the photochromic mechanism is caused by the ring-closure reaction. In addition, compound TrPEF_2_-MA displayed an excellent reversibility during the photochromic process, which shows no obvious fatigue even after 20 photochromic and bleaching cycles (Figure 2(d)). The excellent recyclability and slow thermal-back reaction indicate its huge potential applications as rewritable and reversible photoresponsive materials.

The fact that TrPEF_2_-MA is an oily liquid at room temperature, the photochromic properties of TrPEF_2_-MA films added into polymer matrix were also investigated. 2-mm-thick photochromic films with the doping concentration of 10 wt% in polymer were prepared by photocuring the 3D-printable resin. The preparation details of the polymer films are described in the Supporting Information. Photophysical studies of TrPEF_2_-MA films were listed in Figure S1. The transparent flexible films showed good flexibility and photochromic properties. Upon UV light irradiation for ca. 50 seconds, the polymer films changed from colorless to yellow; simultaneously, the emission properties of the polymer films were quenched. Similar to the TrPEF_2_-MA in solution, a reverse process occurred when irradiated by visible light. The UV-Vis absorption spectra as a function of the photochromic bleaching time in films are shown in Figure S5. It can be clearly seen that the films faded within 2310 seconds upon visible light irradiation. Compared with the solution state (THF), the absorption bands of the doped film were slightly red-shifted to 482 nm, which is mainly due to the environmental constraints in the rigid polymer matrix [58]. The light transmittance of this film reached over 80% in daylight, while drastic decrement was observed in the range of 400 to 600 nm when exposed to UV light, as shown in Figure 2(e). The distinctive transmitting properties of the polymer films provide numerous potential applications, such as photoswitchable patterning and anticounterfeiting applications in solid surface and interface [59]. Moreover, the stability of the doped and copolymerized photoresponsive polymer samples was investigated as shown in Figure 2(f). The fabrication procedure of polymer films was described in details in the Supporting Information [51]. After soaking polymer films in THF solution overnight, the photochromic behavior of the doped polymer films disappeared, while the copolymerized films retained (Figure 2(g)). It reveals that the photoresponsive functional groups in doped polymer film are unstable when dissolved in organic solvent. In copolymerized films, the grafted triphenylethylene groups are much more stable under organic solvent corrosion. Moreover, the differential scanning calorimetry (DSC) and thermal gravimetric analyses (TGA) studies were carried out to study thermal properties of photoresponsive polymers (Figure S2 and S3). The copolymerized film showed a slightly higher degrading temperature (358°C) than that of the doped polymer film (271°C), indicating its better thermal stability. It could be ascribed to the strong covalent bonds between the cross-linking network. In addition, the scanning electron microscopy (SEM) studies of the two films were conducted as shown in Figures 2(h) and 2(i). It can be clearly seen that TrPEF_2_-MA can be more uniformly dispersed in the polymer matrix by copolymerization than direct doping. Hence, it could be concluded that the fabrication of photoresponsive 3D structures based on directly printable photoresponsive resins showed greater advantages both in thermal and chemical stabilities than those doped photoresponsive resins.

### 2.2. Constructions of Photoresponsive 3D Structures

**Figure 3:**
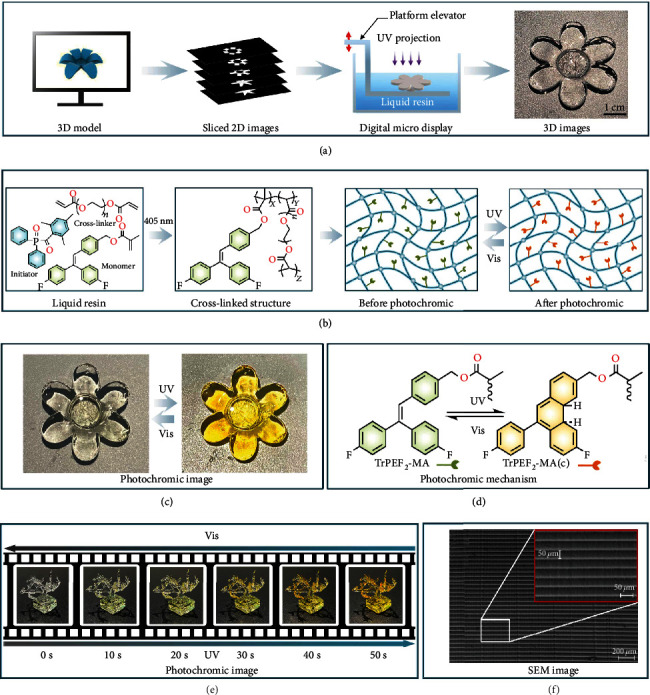
Constructions and the investigation of photoresponsive 3D structures. (a) Schematic of digital light processing-based 3D printing process. (b) Chemical structures of photopolymer inks (including monomer, cross-linker, and initiator) and formed cross-linked structure via photopolymerization and the general schemes of dynamic-based photochromic covalent bonds before and after UV/Vis irradiation. (c) Images of 3D-printed photoresponsive flower. (d) Schematic diagram of photochromic mechanism of triphenylethylene group. (e) Photochromic process of the 3D-printed tree based on TrPEF_2_-MA containing resin. (f) SEM images of the printed photoresponsive 3D structures.

To fabricate various photoresponsive 3D structures, the photochromic liquid resins were directly used in DLP 3D printing process as shown in Figure 3(a). By adding different ratios of the photoresponsive molecule TrPEF_2_-MA in PEGDA (cross-linker) [60, 61] and TPO (initiator) [62], four types of 3D-printable liquid resins, resin A (without TrPEF_2_-MA), resin B (with 5 wt% TrPEF_2_-MA), resin C (with 10 wt% TrPEF_2_-MA), and resin D (with 20 wt% TrPEF_2_-MA), were prepared, and the details were listed in Table S1.

As illustrated in Figure 3(b), the solidified 3D structures were macromolecular-based networks, which were composed of cross-linked PEGDA and grafted TrPEF_2_-MA functional groups. The copolymerized chemical structures were further verified by the Fourier transform infrared (FTIR) spectra as shown in Figure S4. With 3D-printable liquid resin C, a simple “light-responsive flower” 3D structure was fabricated (Figures 3(a) and 3(c)). This transparent “light-responsive flower” could gradually turn to bright yellow upon UV irradiation (50 seconds) and recover to transparent by visible light irradiation (2310 seconds). According to previous results, the photochromic property of the 3D printing structure could be ascribed to the ring-closure reaction of the TrPEF_2_-MA functional groups as shown in Figure 3(d) [51]. Likewise, a more complex 3D structure “light-responsive tree” was also printed with the same resin as shown in Figure 3(e). The specific printing parameters and operational details are presented in Table S2. This “light-responsive tree” gradually turned from transparent to orangish yellow within 50 seconds under 365-nm UV light. Thus, the color saturations of the photoresponsive 3D structures could be precisely manipulated by adjusting the UV irradiation time. In addition, SEM studies of the printed 3D structures were performed as shown in Figure 3(f). All showed uniform layers with the thickness of *ca.* 50 *μ*m, which is consistent with the preset slice thickness, indicating the high fidelity during the DLP 3D printing process.

**Figure 4:**
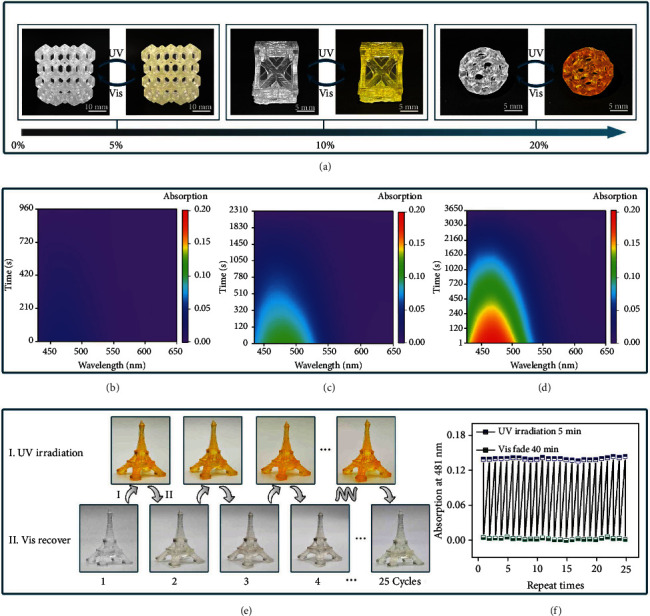
Photochromic properties of the printed 3D structures. (a) Photoresponsive pictures of the printed hollow 3D structures containing different mass fractions of TrPEF_2_-MA. (b) Time-dependent UV-Vis absorption spectra of the printed hollow 3D structures with resin B during the photochromic bleaching process. (c) Time-dependent UV-Vis absorption spectra of the printed hollow 3D structures with resin C during the photochromic bleaching process. (d) Time-dependent UV-Vis absorption spectra of the printed hollow 3D structures with resin D during the photochromic bleaching process. (e and f) Recycling of the photochromic process of 3D-printed Eiffel Tower as a function of exposure to UV light (365 nm) and visible light.

One of the most important advances for DLP 3D printing technology is that it can achieve the manufacture of complex hollow structures. A series of high-resolution hollow structures were fabricated with these photoresponsive 3D-printable resins containing different mass fractions of TrPEF_2_-MA. These printed honeycomb structure (resin B), modified hollow cube (resin C), and porous hollow sphere (resin D) showed excellent printing resolutions and smooth surfaces, indicating the excellent performances for these resins in fabricating complex structures (Figure 4(a)). Under sufficient UV irradiations, these 3D hollow structures with different proportions of TrPEF_2_-MA turned to pale yellow (resin B), bright yellow (resin C), and orangish yellow (resin D), respectively, as shown in Figure 4(a).

Moreover, UV/Vis reflectance spectra of these 3D hollow structures were collected. As shown in Figures 4(b)–4(d), the absorption maxima of resins B, C, and D are 468, 482, and 471 nm, respectively. It can be clearly seen that the absorption intensities significantly boosted with the increasing proportions of TrPEF_2_-MA, 0.032 in resin B, 0.108 in resin C, and 0.194 in resin D. Besides, the color maintaining times were prolonged from 960 seconds (resin B) to 3650 seconds (resin D), and the half-life was also extended from 261.5 seconds (resin B) to 866.4 seconds (resin D) as shown in Figure S7–S9. Moreover, through time-dependent UV-Vis absorption test at different temperatures, it was found the half-life of photochromism shortened with the increase of temperature as shown in Figure S10–S13. Thus, the color saturations and maintaining times of the photoresponsive 3D structures could be precisely controlled and easily adjusted by tuning the proportion of TrPEF_2_-MA in resins and temperature. In addition, all these complex hollow structures showed excellent printing resolutions and smooth surfaces, indicating the excellent stability for these resins during DLP 3D printing. Furthermore, the 3D hollow structures also displayed an excellent photochromic reversibility. After 25 photochromic and bleaching cycles, insignificant fatigue was detected (Figures 4(e)–4(f) and Movie S1). These superiorities of TrPEF_2_-MA indicate the potential applications in information hiding, information carrying, and others (Figure S14).

### 2.3. Multicomponent Photoresponsive 3D Structures

**Figure 5:**
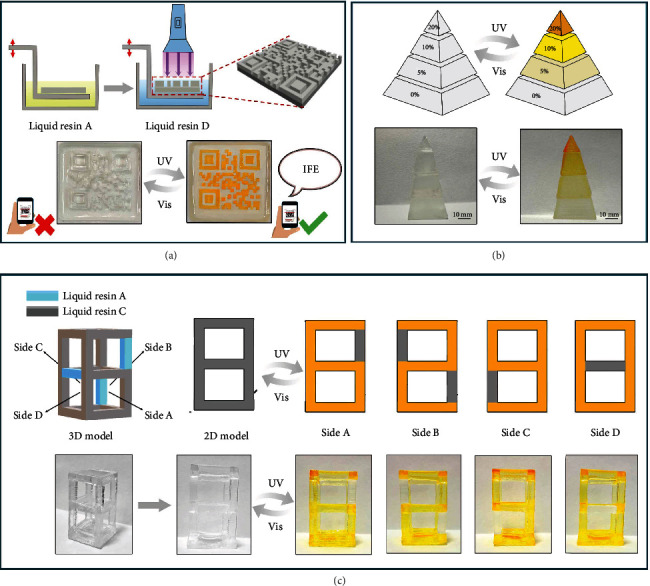
3D-printed multicomponent photoresponsive structures. (a) Schematic of 3D-printed multicomponent QR code with liquid resin A and resin D. (b) A printed photochromic four-tier pyramid with resins A-D. (c) Schematic of the 3D-printed multicomponent framework for information carrying and encryption.

To clearly illustrate the potential applications of the photoresponsive 3D structures, multicomponent 3D printing processes were also performed with different resins to realize information hiding and information carrying in 3D structures. Firstly, a double-layered QR code structure was printed by automatically switching liquid resins during the printing process as shown in Figure 5(a) and Movie S2. The top layer (printed by liquid resin D) in the QR pattern was combined with a bottom plate (printed by liquid resin A). The printed double-layered QR code was transparent before UV irradiation, which could use for information hiding. Under UV light for 60 seconds, the top layer QR matrix was changed from transparent to yellow, which could be directly scanned with a cellphone to get the information “IFE.” The scannable QR code could be reversibly turned to transparent within 2310 seconds under visible light irradiation. In addition, a four-tier pyramid was printed with different resins containing TrPEF_2_-MA of 0, 5, 10, and 20 wt% from the bottom to the top, respectively (Figure 5(b)), as the color saturations of the photochromic 3D structure could be precisely tuned by manipulating the proportion of TrPEF_2_-MA in the resins. The photoreponsive four-tier pyramid altered to gradient-yellow upon UV light and recovered to transparent under visible light. The photoresponsive gradient-color 3D structures are quite striking in adaptive camouflage. As shown in Figure 5(c), a transparent framework was printed with resins A (0 wt% TrPEF_2_-MA) and C (10 wt% TrPEF_2_-MA) to realize information carrying and encryption. By rational arrangement of printing resins for the framework, information could be successfully hidden in the 3D structure. By irradiating the 3D framework with UV light, components printed with resin C turned to yellow, while the remaining component maintained transparent. Thus, four different numbers 6, 2, 9, and 0 appeared on each surface of the framework and disappeared under visible light irradiation. According to this demonstration, key information could be carried or hidden in the designed 3D structures. Therefore, the development of these new photoresponsive 3D-printable resins paves a new way to endow a wide variety of practical functions such as advanced anticounterfeiting, information hiding or carrying, and adaptive camouflage to traditional printed 3D structures.

## 3. Conclusion

In summary, we have proposed a rational strategy to construct photoresponsive prescribed complex 3D structures. By attaching the UV curable MA group to photochromic triphenylethylene, a photoresponsive material TrPEF_2_-MA that can be used for directly DLP 3D printing was realized. A series of photoresponsive 3D structures with high resolution (up to 50 *μ*m) were fabricated. These 3D structures showed a striking color switching between transparent and yellow under alternating UV/visible light irradiation with good repeatability. To illustrate the information hiding and information-carrying properties, multicomponent DLP 3D printing processes were employed. Information hiding (QR codes), multicolor photoresponsive, and information-carrying multimaterial-based 3D structures were successfully printed. This work provides a novel and efficient strategy to construct photoresponsive 3D structures for information hiding and paves the way for more potential applications in adaptive camouflage, information storage, and flexible electronics.

## Data Availability

All relevant data that support the findings are available within this article and supporting information and are also available from authors upon reasonable request.

## Supplementary Materials

Supplementary Materials Materials and methods, characterization data, emission spectra, TG analysis, DSC curves, absorption spectra of film, FT-IR spectra spectroscopy, and movies of 3D printing photoresponsive materials.
