# Supplementary material for: Construction of Photoresponsive 3D Structures Based on Triphenylethylene Photochromic Building Blocks
Source: Research (Wash D C). 2022 Sep 2;2022:9834140. doi: 10.34133/2022/9834140 (PMC9484832; doi:10.34133/2022/9834140)
Supplement: Supplementary Materials — Materials and methods, characterization data, emission spectra, TG analysis, DSC curves, absorption spectra of film, FT-IR spectra spectroscopy, and movies of 3D printing photoresponsive materials. [file 9834140.f1.zip › Supporting Information-clean.docx]

Supporting Information

Construction of Photoresponsive 3D Structures Based on Triphenylethylene Photochromic Building Blocks

Xiayu Zhang^†, 1^, Fukang Liu^†, 1^, Beibei Du^1^, Rongjuan Huang^1^, Simin Zhang^1^, Yunfei He^1^, Hailan Wang^1^, Jingjing Cui^1^, Biao Zhang*^1^, Tao Yu*^1^, Wei Huang*^1, 2, 3^

1. Frontiers Science Center for Flexible Electronics, Shaanxi Institute of Flexible Electronics & Shaanxi Institute of Biomedical Materials and Engineering, Northwestern Polytechnical University, 127 West Youyi Road, Xi’an 710072, P. R. China.

2. Key Laboratory of Flexible Electronics & Institute of Advanced Materials, Nanjing Tech University, 30 South Puzhu Road, Nanjing 211816, P. R. China.

3. Key Laboratory for Organic Electronics and Information Displays & Institute of Advanced Materials, Nanjing University of Posts and Telecommunications, Nanjing 210023, P. R. China.

E-mail: iambzhang@nwpu.edu.cn; iamtyu@nwpu.edu.cn; iamwhuang@nwpu.edu.cn

†These authors contribute equally to this work.

CONTENT

[Experimental Procedures 3](#_Toc109554375)

[Materials 3](#_Toc109554376)

[Synthesis of photochromic compounds 3](#_Toc109554377)

[Scheme S1. Synthetic routes for TrPEF_2_-MF, TrPEF_2_-A and TrPEF_2_-MA. 3](#_Toc109554378)

[Synthesis of ethyl 4-(2,2-bis(4-fluorophenyl)vinyl)benzoate (TrPEF_2_-MF) 3](#_Toc109554379)

[Synthesis of (4-(2,2-bis(4-fluorophenyl)vinyl)phenyl)methanol (TrPEF_2_-A) 4](#_Toc109554380)

[Synthesis of 4-(2,2-bis(4-fluorophenyl)vinyl)benzyl methacrylate (TrPEF_2_-MA) 4](#_Toc109554381)

[Synthesis of dehydrogenated photoisomer TrPEF_2_-MA(O) 4](#_Toc109554382)

[Preparation of photochromic liquid resin 4](#_Toc109554383)

[Preparation method of copolymerized films 5](#_Toc109554384)

[3D printing fabrication 5](#_Toc109554385)

[Characterization 5](#_Toc109554386)

[Table S1. Material used in the photochromic liquid resin. 5](#_Toc109554387)

[Table S2. Photoresponsive 3D structures printing parameters. 6](#_Toc109554388)

[Supplementary Information 6](#_Toc109554389)

[Figure S1. Normalized emission spectrum of photoresponsive 3D structures. 6](#_Toc109554390)

[Figure S2. TG analysis of solidified polymer with different liquid resin after polymerization. 7](#_Toc109554391)

[Figure S3. DSC curves of solidified polymer with different liquid resin after polymerization. 7](#_Toc109554392)

[Figure S4. FTIR spectrum of solidified polymer with different liquid resin after polymerization. 8](#_Toc109554393)

[Figure S5. Time-dependent UV-vis absorption spectra of TrPEF_2_-MA film. 8](#_Toc109554394)

[Figure S6. Plot of ln(A/A_0_) versus time for the absorbance decay of TrPEF_2_-MA 9](#_Toc109554395)

[Figure S7. Plot of ln(A/A_0_) versus time for the absorbance decay of liquid resins B. 9](#_Toc109554396)

[Figure S8. Plot of ln(A/A_0_) versus time for the absorbance decay of liquid resins C 10](#_Toc109554397)

[Figure S9. Plot of ln(A/A_0_) versus time for the absorbance decay of liquid resins D 10](#_Toc109554398)

[Figure S10. Time-dependent UV-vis absorption spectra of TrPEF_2_-MA film at 273 K 11](#_Toc109554399)

[Figure S11. Plot of ln(A/A_0_) versus time for the absorbance decay of liquid resins D at 273 K. 11](#_Toc109554400)

[Figure S12. Time-dependent UV-vis absorption spectra of TrPEF_2_-MA film at 323 K. 12](#_Toc109554401)

[Figure S13. Plot of ln(A/A0) versus time for the absorbance decay of liquid resins D at 323 K. 12](#_Toc109554402)

[Figure S14. A series of images sequentially write into and erase from the TrPEF_2_-MA film. 13](#_Toc109554403)

[Characterization of chemical structure 13](#_Toc109554404)

[Figure S15. ^1^H NMR spectrum of TrPEF_2_-MF (in Chloroform-*d*). 13](#_Toc109554405)

[Figure S16. ^1^H NMR spectrum of TrPEF_2_-A (in Chloroform-*d*). 14](#_Toc109554406)

[Figure S17. ^1^H NMR spectrum of TrPEF_2_-MA (in Chloroform-*d*). 14](#_Toc109554407)

[Figure S18. ^1^H NMR spectrum of TrPEF_2_-MA(O) (in Chloroform-*d*). 15](#_Toc109554408)

[Figure S19. High resolution Mass spectrum of TrPEF_2_-MF. 15](#_Toc109554409)

[Figure S20. High resolution Mass spectrum of TrPEF_2_-A. 16](#_Toc109554410)

[Figure S21. High resolution Mass spectrum of TrPEF_2_-MA. 16](#_Toc109554411)

[Reference 16](#_Toc109554412)

Experimental Procedures

Materials

Methyl 4-(Bromomethyl)Benzoate, Triethyl phosphite, 4,4'-Difluorobenzophenone, Potassium Tert-Butoxide (t-BuOK), Diisobutylaluminium hydride (DIBAL-H), Methacryloylchloride, Triethylamine, Magnesium sulfate, n-Hexane, Ethanol, Dichloromethane (CH_2_Cl_2_), Tetrahydrofuran (THF), α,ω-Diacryloyl Poly(Ethylene Glycol) (Mn=700) (PEGDA), Diphenyl(2,4,6-trimethylbenzoyl)phosphine oxide (TPO). All regents and solvents were purchased from Aladdin, Titan or Adamas, and were used as received.

Synthesis of photochromic compounds

Synthetic routes for TrPEF_2_-MF, TrPEF_2_-A and TrPEF_2_-MA were described in Scheme S1. The intermediate compounds benzoic acid,4-[(diethoxyphosphinyl)methyl],ethyl ester and TrPEF_2_ were synthesized in accordance with the previous literatures^[1-2]^. All the products were purified by column chromatography and were confirmed by ^1^H NMR spectroscopy, high-resolution EI mass spectroscopy.

Scheme S1. Synthetic routes for TrPEF_2_-MF, TrPEF_2_-A and TrPEF_2_-MA.

Synthesis of ethyl 4-(2,2-bis(4-fluorophenyl)vinyl)benzoate (TrPEF_2_-MF)

To a two-necked round-bottomed flask containing 4,4'-difluorobenzophenone (4.36 g, 19.98 mmol) and benzoic acid,4-​[(diethoxyphosphinyl​)​methyl],ethyl ester (5.00 g, 16.65 mmol) was added degassed THF (70 mL) under an argon atmosphere. After cooling to 0 °C, t-BuOK (4.67 g, 41.63 mmol) was added. Upon stirring for 12 hours under an argon atmosphere, the mixture was poured into ethanol/ deionized water (250 ml, v/v = 3:2), and stirred for another 1 hour. The white precipitate which formed was collected by filtration. The precipitate was dissolved in CH_2_Cl_2_ and washed 3 times with water. The organic layer was dried over anhydrous MgSO_4_. After evaporation of the filtrate, the residue was purified by recrystallization via vapor diffusion of hexane into a concentrated dichloromethane solution of the product to give TrPEF_2_-MF as a white powder. Yield: 4.20 g (69.22 %). ^1^H NMR (500 MHz, Chloroform-*d*) δ (TMS, ppm): δ = 1.35-1.38 (t, J = 7.0 Hz, 3H), 4.62-4.36 (q, J = 7.5 Hz, 2H), 6.91 (s, 1H), 7.00-7.07 (m, 6H), 7.11-7.14 (d, J = 5.5 Hz, 2H), 7.27-7.29 (d, J = 5.5 Hz, 2H), 7.75-7.27 (d, J = 8.5 Hz, 1H), 7.81-7.83 (d, J = 8.0 Hz,1H); High solution EI-MS: m/z found: 364.1269 [M]+; calcd for C_23_H_18_F_2_O_2_: 364.1275.

Synthesis of (4-(2,2-bis(4-fluorophenyl)vinyl)phenyl)methanol (TrPEF_2_-A)

To a solution of ethyl 4-(2,2-bis(4-fluorophenyl)vinyl)benzoate (4.00 g, 10.98 mmol) in dry CH_2_Cl_2_ (100 mL) cooled to 78 °C with stirring under nitrogen was added dropwise a solution of DIBAL-H (47 mL,1.0 M solution in n-hexane). Stirring was continued for 4 h at 78 °C and the reaction mixture was then allowed to warm to 0 °C and quenched with H_2_O. The organic layer was separated and the aqueous was extracted with CH_2_Cl_2_. The combined organic extracts were dried over MgSO_4_ and evaporated to yield quantitatively the desired alcohol as a white solid. Yield: 3.10 g (87.61 %). ^1^H NMR (500 MHz, Chloroform-*d*) δ (TMS, ppm): δ = 4.63 (s, 1H), 5.30 (s, 1H), 6.89 (s, 1H), 6.99-7.04 (m, 6H), 7.14-7.17 (m, 4H), 7.26-7.28 (m, 2H); High solution EI-MS: m/z found: 322.1161 [M]+; calcd for C_21_H_16_F_2_O: 322.1169.

Synthesis of 4-(2,2-bis(4-fluorophenyl)vinyl)benzyl methacrylate (TrPEF_2_-MA)

To methacryloylchloride (500.00 mg 4.78 mmol) in CH_2_Cl_2_ (100 mL) was added (4-(2,2-bis(4-fluorophenyl)vinyl)phenyl)methanol (1.54 g, 4.78 mmol) and Et3N (968.05 mg, 9.57 mmol) at 0 °C. The reaction was stirred overnight, and then quenched with aqueous 1 M HCl. The phases were separated and the aqueous phase was extracted with CH_2_Cl_2_ (× 3). The combined organic extracts were dried over MgSO_4_ and concentrated in vacuo. Chromatography on a silica-gel column with dichloromethane-hexane (1/5, v/v) as eluent was then performed for the further purification to get transparent oily liquid of TrPEF_2_-MA (1.15 g, 2.95 mmol) with 61.58% yield. ^1^H NMR (500 MHz, Chloroform-*d*) δ (TMS, ppm): δ = 1.96 (s, 3H), 5.12 (s, 2H), 5.59 (s, 1H), 6.15 (s, 1H), 6.89 (s, 1H), 6.99-7.05 (m, 6H), 7.14-7.17 (m, 4H), 7.25-7.28 (m, 2H); High solution EI-MS: m/z found: 390.1422 [M]+; calcd for C_25_H_20_F_2_O_2_: 390.1431.

Synthesis of dehydrogenated photoisomer TrPEF_2_-MA(O)

A mixture of 4-(2,2-bis(4-fluorophenyl)vinyl)benzyl methacrylate (TrPEF_2_-MA) (100.0 mg, 0.26 mmol) in n-Hexane(10 ml) was irradiated with UV-light in a vial under oxygen for 12 h. The mixture was dissolved in 20 ml dichloromethane and washed with MgSO_4_ aqueous solution for 3 times. The mixture was further purified by column chromatography with n-hexane as eluent. A white solid named TrPEF_2_-MA(O) was obtained. Yield: 85.1 mg (85.4 %). ^1^H NMR (500 MHz, Chloroform-*d*) δ (TMS, ppm): δ = 2.02 (s, 3H), 5.47 (s, 2H), 5.63 (s, 1H), 6.22 (s, 1H), 7.19-7.23 (t, J = 8.5 Hz, 2H), 7.28-7.32 (m, 1H), 7.46-7.49 (d, J = 5.5 Hz, 2H), 7.61 (s, 1H), 7.65-7.67 (d, J = 7.0 Hz, 1H), 7.83-7.85 (d, J = 5.5 Hz, 1H), 7.89-7.90 (d, J = 8.0 Hz, 1H), 8.35-8.38 (d, J = 2.5 Hz, 1H), 8.58 (s, 1H).

**Scheme S2.** Cyclization reaction in the photochromism process of TrPEF_2_-MA.

Preparation of photochromic liquid resin

The liquid resins were prepared by mixing PEGDA, TrPEF_2_-MF and TrPEF_2_ with different weight fractions, followed by mechanical stirring for 3 h to make the resins uniform and stable. Then, we added TPO as free radical photoinitiator at a rate of 1.0 wt%, which dispersed evenly and stably in this mixed system. After all ingredients were mixed, the mixtures were conducted by using an ultrasonic bath for 30 min and degassing in a vacuum oven for 30 min. Once finished, the liquid resins were well prepared for following 3D printing.

Preparation method of copolymerized films

Take 2ml of liquid resin with a pipette and pour it into the Teflon mold. Then, the liquid resin was irradiated with a 405 nm light source every 10 seconds for 20 seconds each time. After 10 times of irradiation, it was cured to form the film.

**Scheme S3.** The reaction equation for the grafting of TrPEF_2_-MA on PEGDA.

3D printing fabrication

The printing system was the self-building DLP 3D printer, of which the light source is 405 nm, was used in this study. The computer design 3D structure were sliced to 2D images according to the required layer thickness with CreationWorkshop, and the printing parameters were set by using the custom LabVIEW. We accomplished 3D printing by using a digital light process (DLP) 3D printing equipment with x-y axis resolution of 39 μm. When printing, the patterned light modulated by digital micro display (DMD), and illuminated onto the surface of photocurable liquid resin to solidify solution layer by layer until entire structure was fabricated. After printing, the obtained structure was sonicated with ethanol to remove uncured oligomer followed by a second stage cure in UV oven (Table S2).

Characterization

^1^H NMR for the materials were performed on a Bruker Avance NEO 500 Nuclear Magnetic Resonance Spectrometer with Chloroform-*d* as solvent and tetramethylsilane (TMS) as the internal standard. High resolution Mass spectra (MS) were recorded on a Exactive GC high resolution mass spectrometer. UV-vis absorption spectra, photoluminescence(PL) spectra, transmittance spectra were obtained on a UV-vis spectrometer (Hitachi U-3900H), a Hitachi F-7100 fluorescence spectrophotometer and an Ocean Optic QE 65Pro spectrometer with Ocean Optic reflection probes R600-125F. The Fourier Transform infrared (FTIR) spectrum was conducted on a FTIR spectrophotometer (Perkin Elmer Frontier). The differential scanning calorimetry (DSC) and thermal gravimetric (TG) tests were measured by differential scanning calorimetry and thermal gravimetric analyzer (TA600, USA), and the printed samples were kept at the heating rate of 10°C·min^-1^ from 25°C to 600°C. The details of printed structure and distribution of photochromic compounds in polymer were conducted by scanning electron microscopy (SEM, ZEISS sigma500, China). The photochromic reverse reaction uses a xenon lamp source with the emission wavelength range of 400-780nm.

Table S1. Material used in the photochromic liquid resin.

| **Molecular name of the constituent** | | **Weight content of the constituent** | | | | |
| --- | --- | --- | --- | --- | --- | --- |
| PEGDA | 99 wt% | | 94 wt% | 89 wt% | 79 wt% | 89 wt% |
| TrPEF_2_-MA | 0 wt% | | 5 wt% | 10 wt% | 20 wt% | 0 wt% |
| TrPEF_2_ | 0 wt% | | 0 wt% | 0 wt% | 0 wt% | 10 wt% |
| TPO | 1 wt% | | 1 wt% | 1 wt% | 1 wt% | 1 wt% |
| Photochromic liquid resin. | liquid resin A | | liquid resin B | liquid resin C | liquid resin D | liquid resin E |

Table S2. Photoresponsive 3D structures printing parameters.

| **Structures** | **Layer thickness** | **Curing time for each layers** | **Height of descend** | **Waiting time** | **Light indensity** |
| --- | --- | --- | --- | --- | --- |
| Flower | 100 μm | 5 s | 2 mm | 8 s | 6.25 mW·cm^-2^ |
| Tree | 100 μm | 5 s | 2 mm | 8 s |  |
| Ideal form | 50 μm | 1.2 s | 2 mm | 12 s |  |
| Cross unit cell | 50 μm | 3 s | 2 mm | 12 s |  |
| Buckyball | 50 μm | 3.5 s | 2 mm | 12 s |  |
| Eiffel Tower | 50 μm | 3.5 s | 2 mm | 12 s |  |
| Pyramid (0%/5%/10%20%) | 100 μm | 0.5 s/2 s/5 s/7 s | 2 mm | 8 s |  |
| QR code  (resin C / resin A) | 200 μm | 8.5 s/0.8 s | 2 mm | 6 s |  |
| Digital model  (resin D / resin A) | 200 μm | 8.5 s/0.8 s | 2 mm | 6 s |  |

Supplementary Information


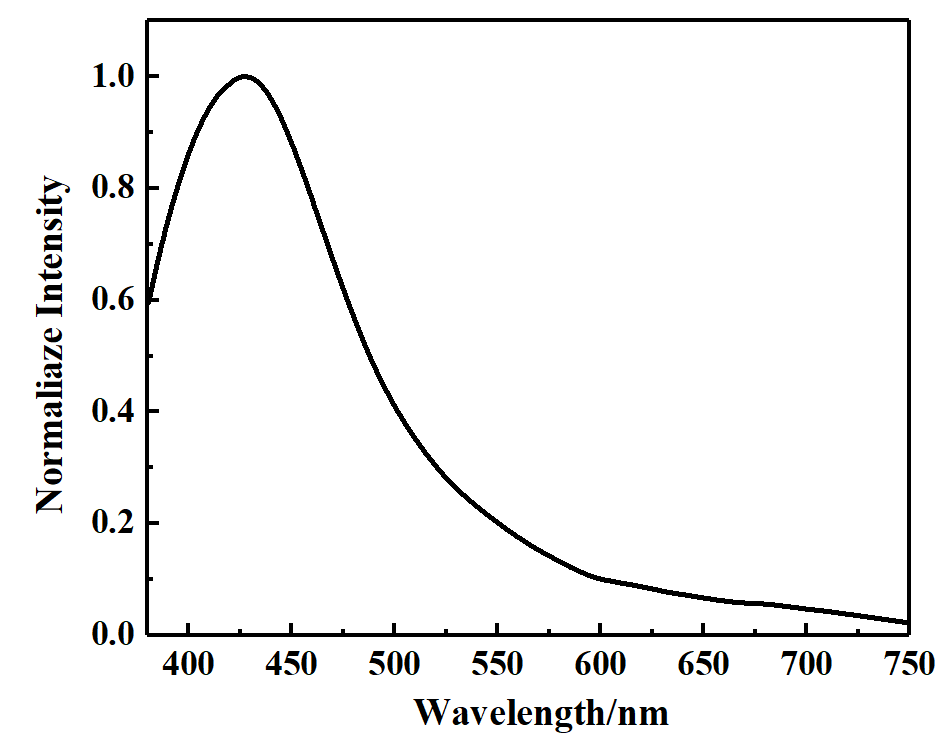


### Figure S1. Normalized emission spectrum of photoresponsive 3D structures.


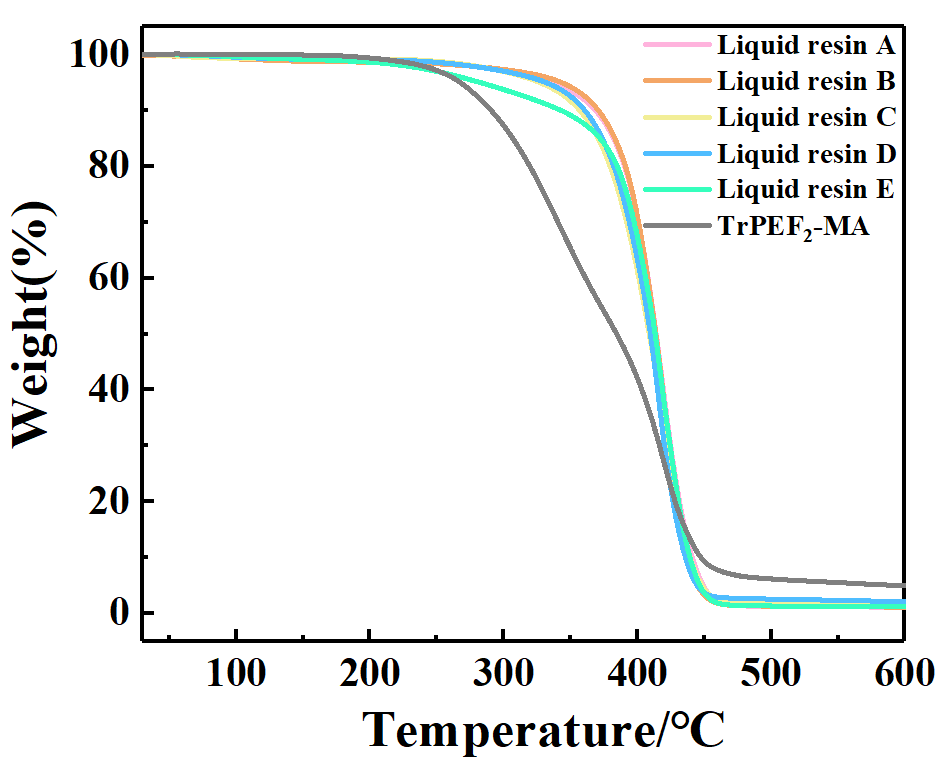


### Figure S2. TG analysis of solidified polymer with different liquid resin after polymerization.


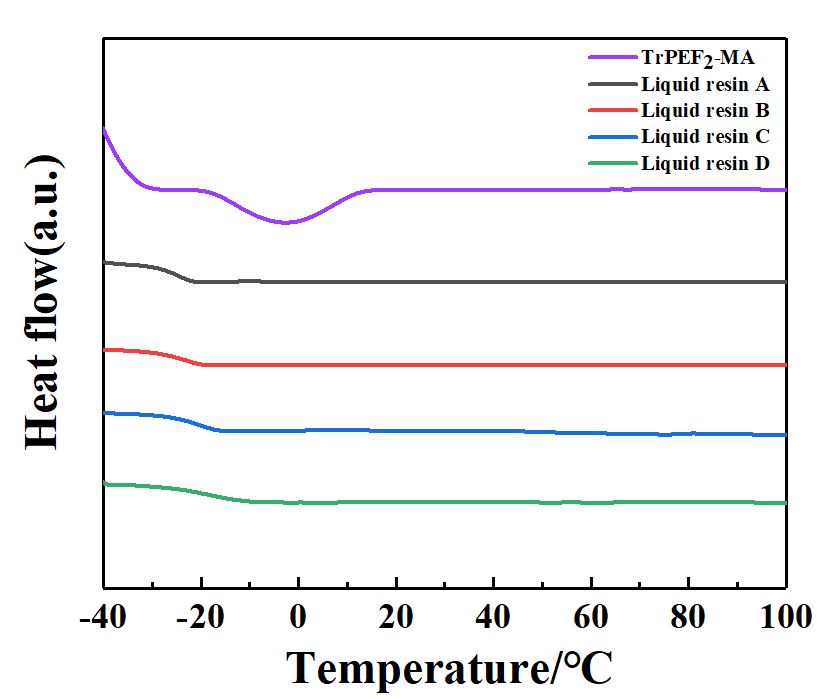


### Figure S3. DSC curves of solidified polymer with different liquid resin after polymerization.


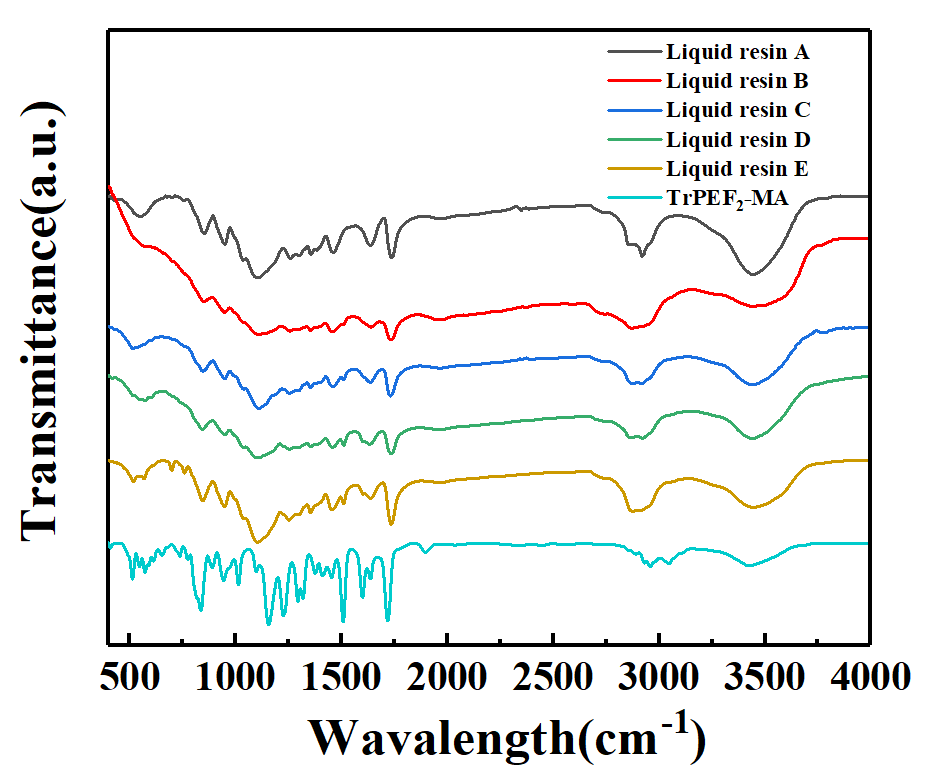


### Figure S4. FTIR spectrum of solidified polymer with different liquid resin after polymerization.


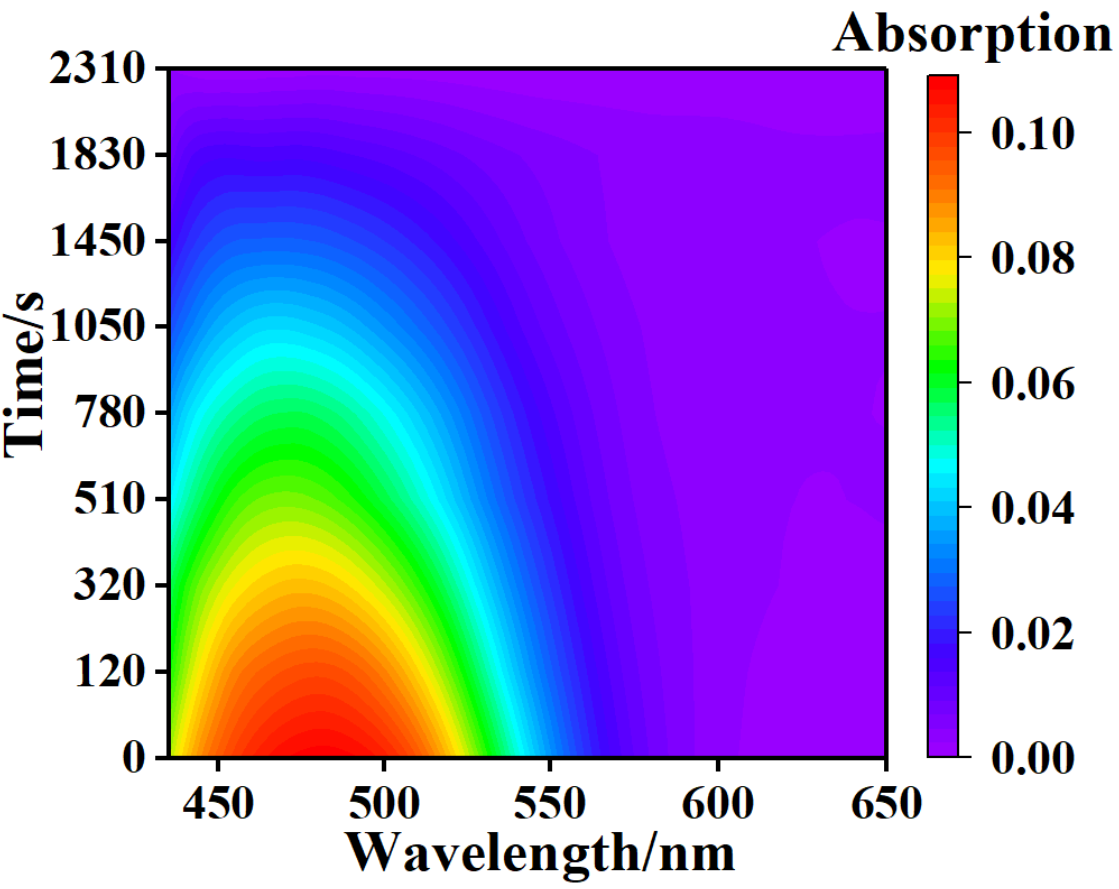


### Figure S5. Time-dependent UV-vis absorption spectra of TrPEF_2_-MA film during the photochromic bleaching process.


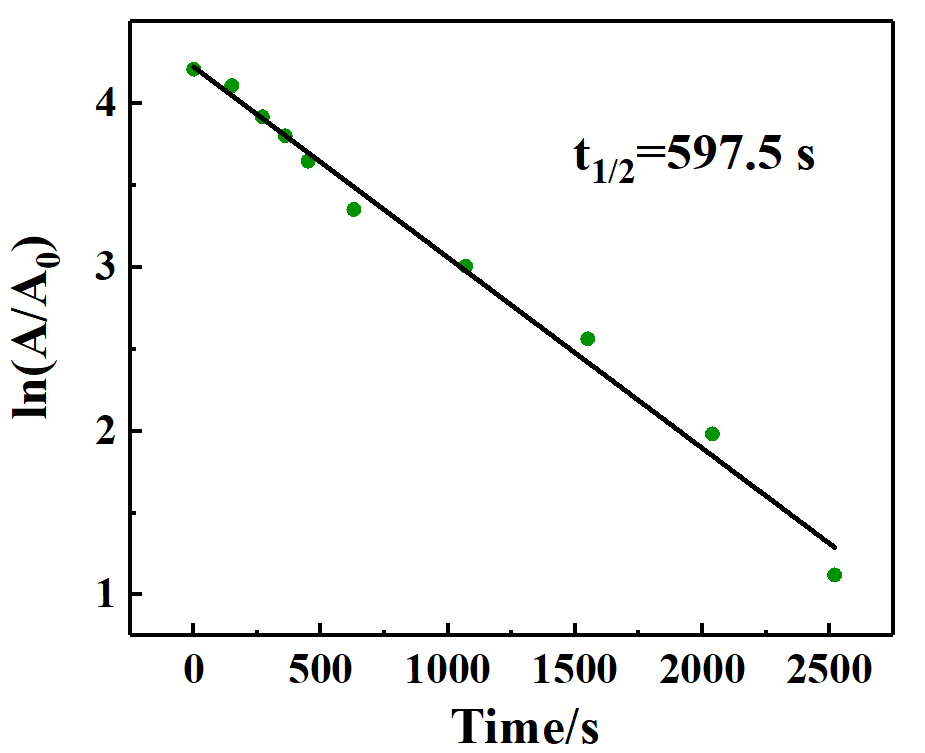


### Figure S6. Plot of ln(A/A_0_) versus time for the absorbance decay of TrPEF_2_-MA at 461 nm at 303 K in degassed in degassed THF solution with concentration of 1.0×10^-1^ M; solid lines represent the theoretical linear fits.


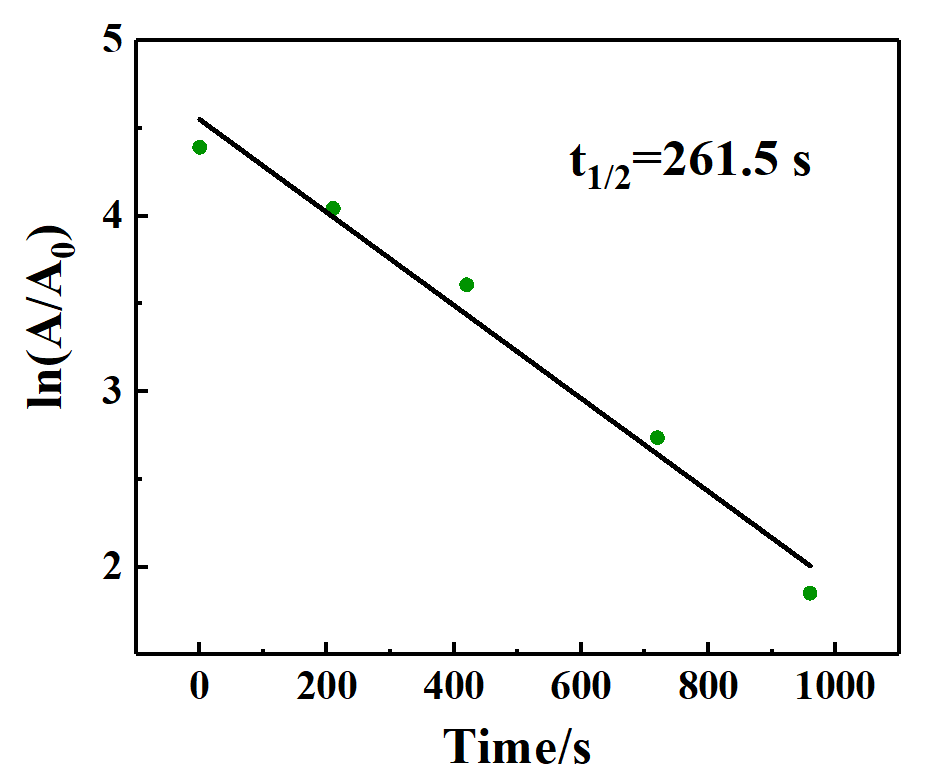


### Figure S7. Plot of ln(A/A_0_) versus time for the absorbance decay of liquid resins B after polymerization at 468 nm at 303 K; solid lines represent the theoretical linear fits.


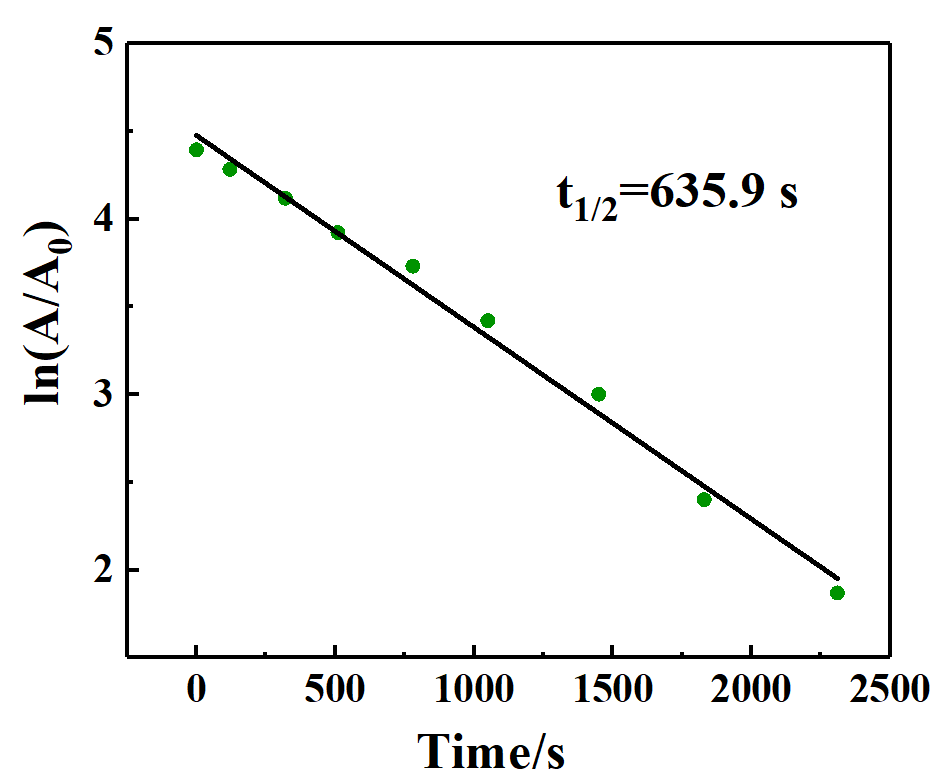


### Figure S8. Plot of ln(A/A_0_) versus time for the absorbance decay of liquid resins C after polymerization at 482 nm at 303 K; solid lines represent the theoretical linear fits.


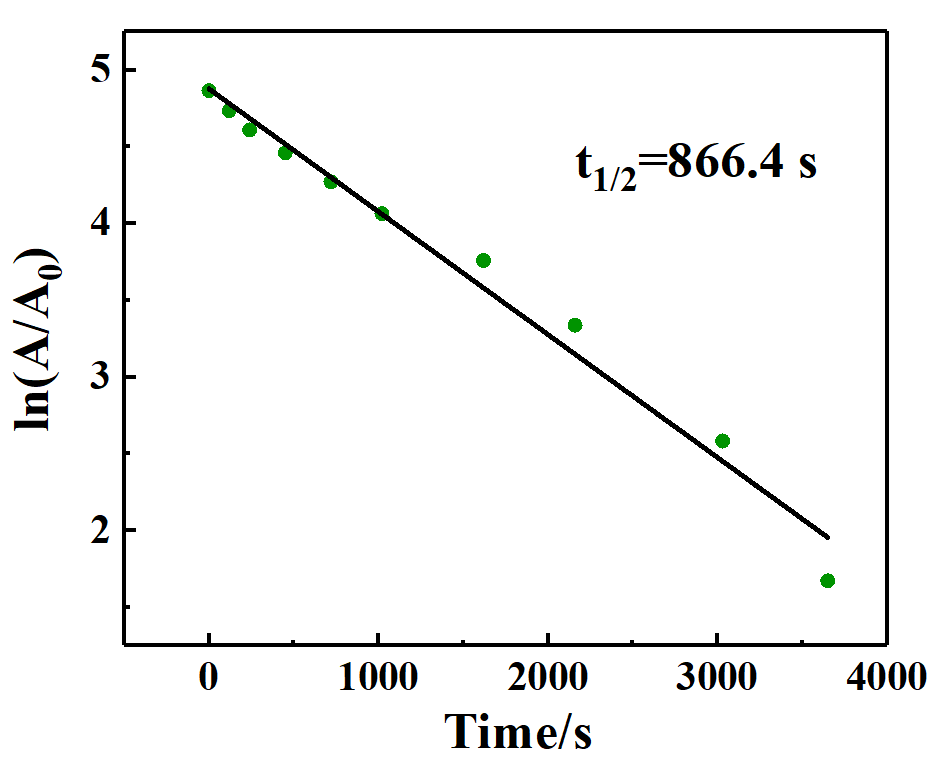


### Figure S9. Plot of ln(A/A_0_) versus time for the absorbance decay of liquid resins D after polymerization at 471 nm at 303 K; solid lines represent the theoretical linear fits.


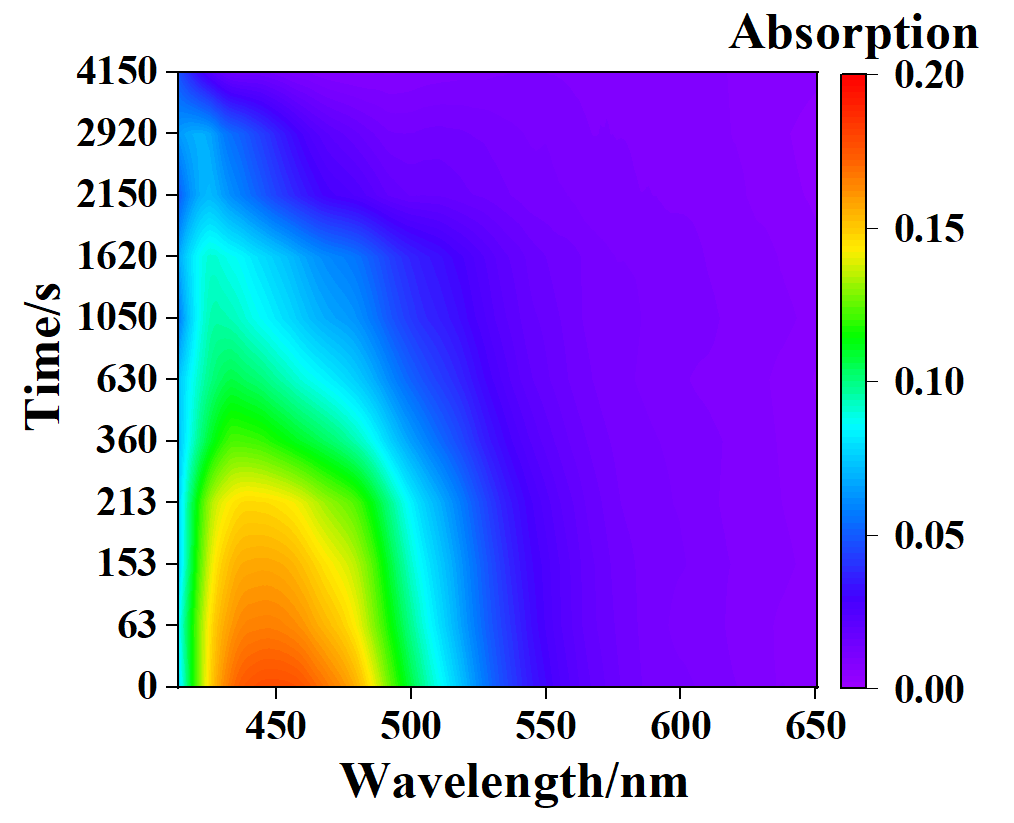


### Figure S10. Time-dependent UV-vis absorption spectra of TrPEF_2_-MA film with liquid resin D during the photochromic bleaching process at 273 K.


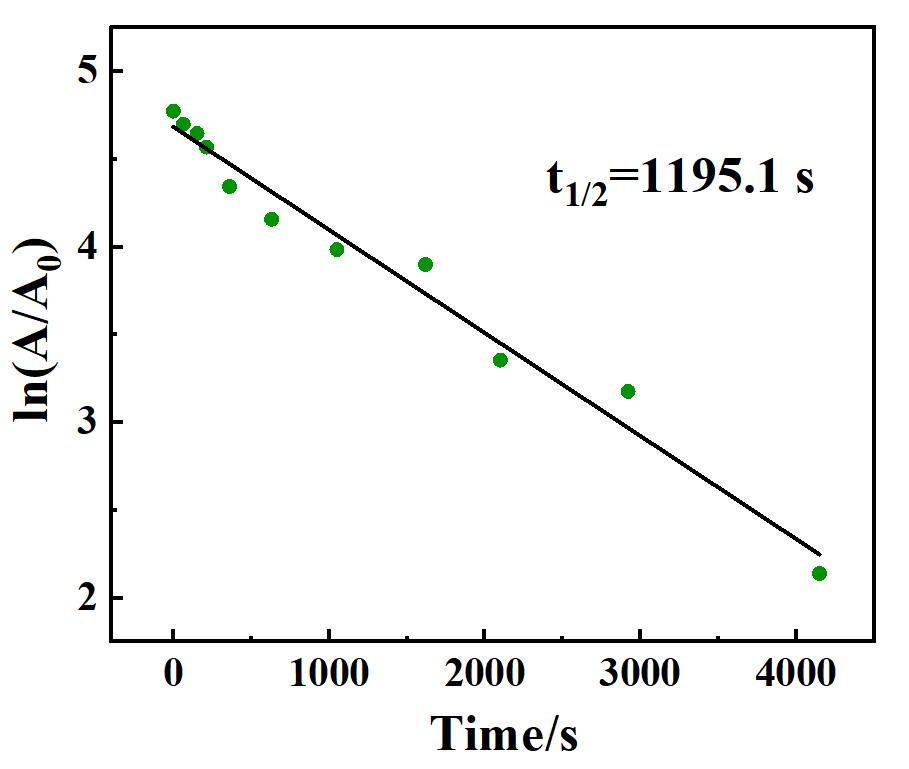


### Figure S11. Plot of ln(A/A_0_) versus time for the absorbance decay of liquid resins D after polymerization at 452 nm at 273 K; solid lines represent the theoretical linear fits.


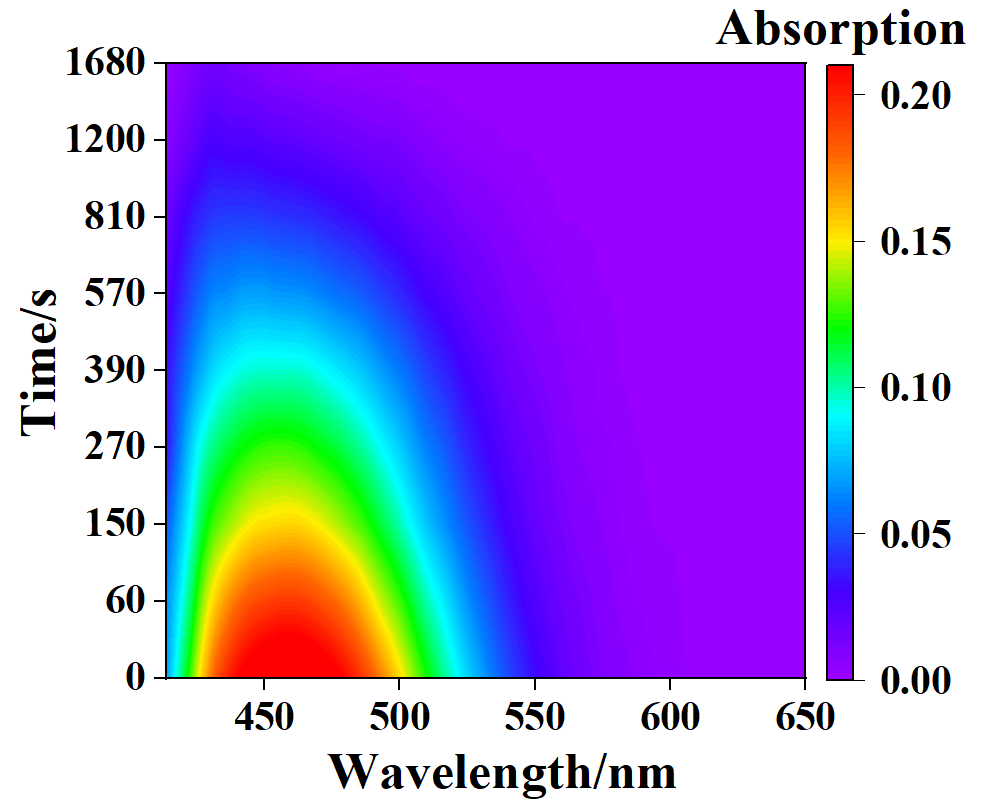


### Figure S12. Time-dependent UV-vis absorption spectra of TrPEF_2_-MA film with liquid resin D during the photochromic bleaching process at 323 K.


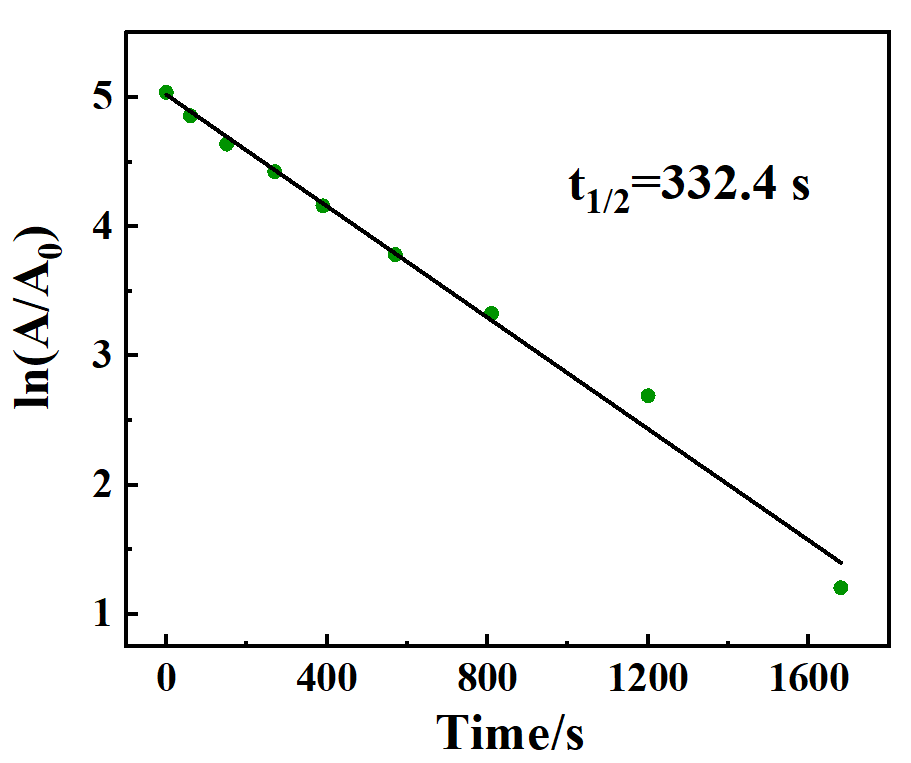


### Figure S13. Plot of ln(A/A_0_) versus time for the absorbance decay of liquid resins D after polymerization at 461 nm at 323 K; solid lines represent the theoretical linear fits.


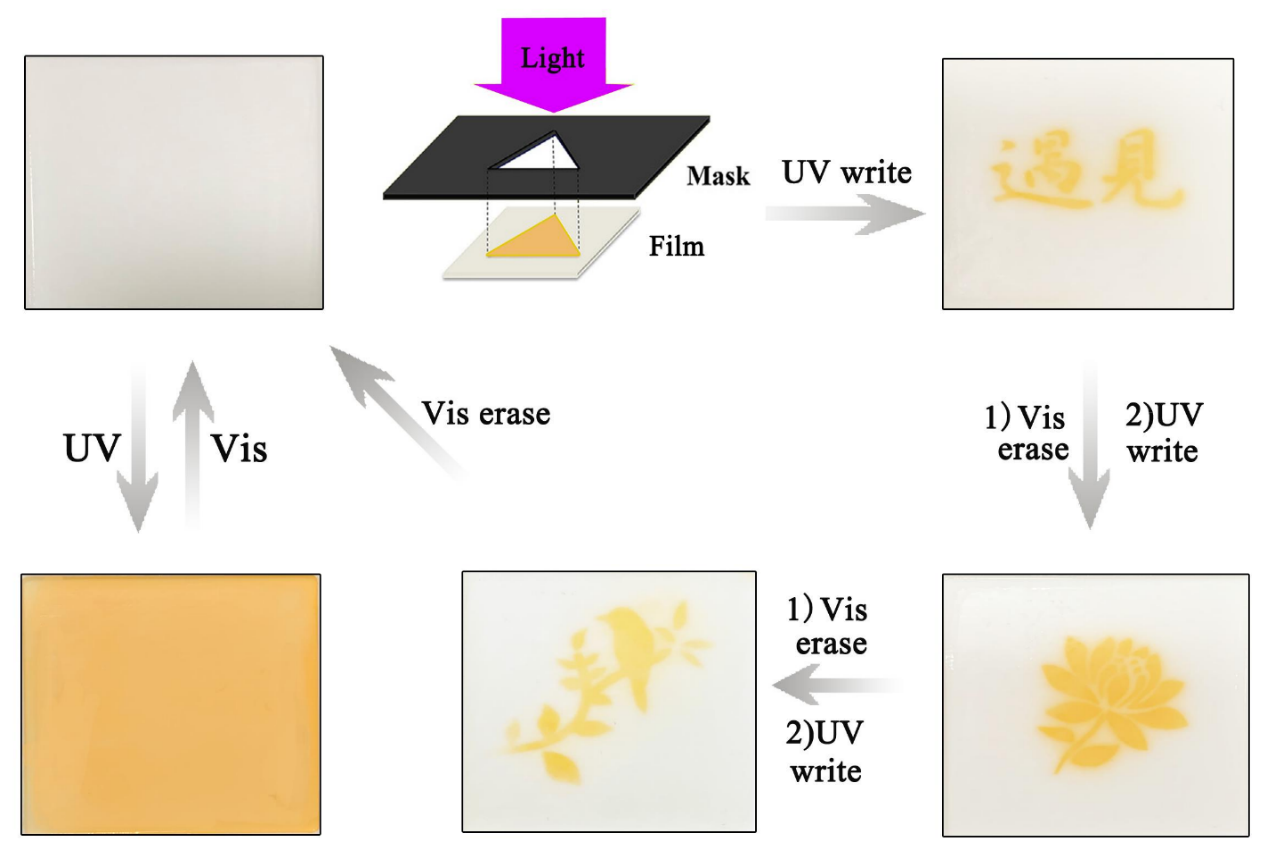


### Figure S14. A series of images sequentially write into and erase from the TrPEF_2_-MA film. The writing time was 2 minutes with 5 W UV lamp. Images were erased by the film was exposed to visible light for 30 minutes.

Characterization of chemical structure


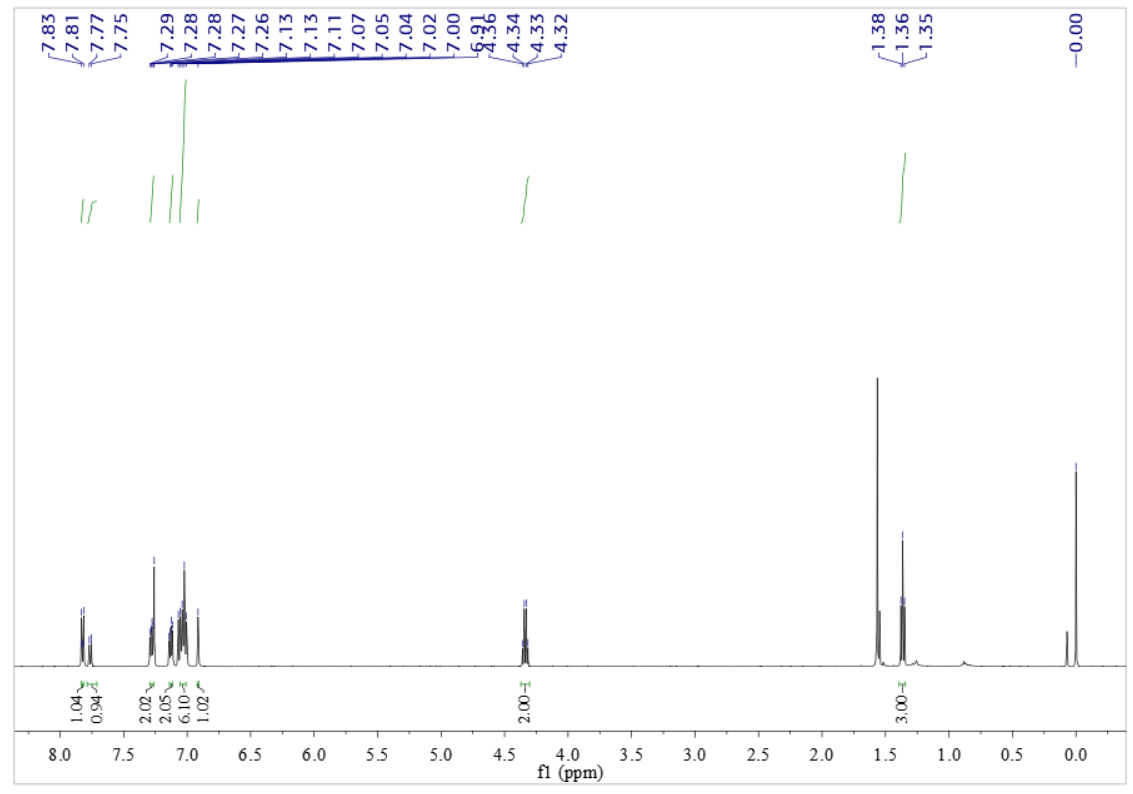


### Figure S15. ^1^H NMR spectrum of TrPEF_2_-MF (in Chloroform-*d*).


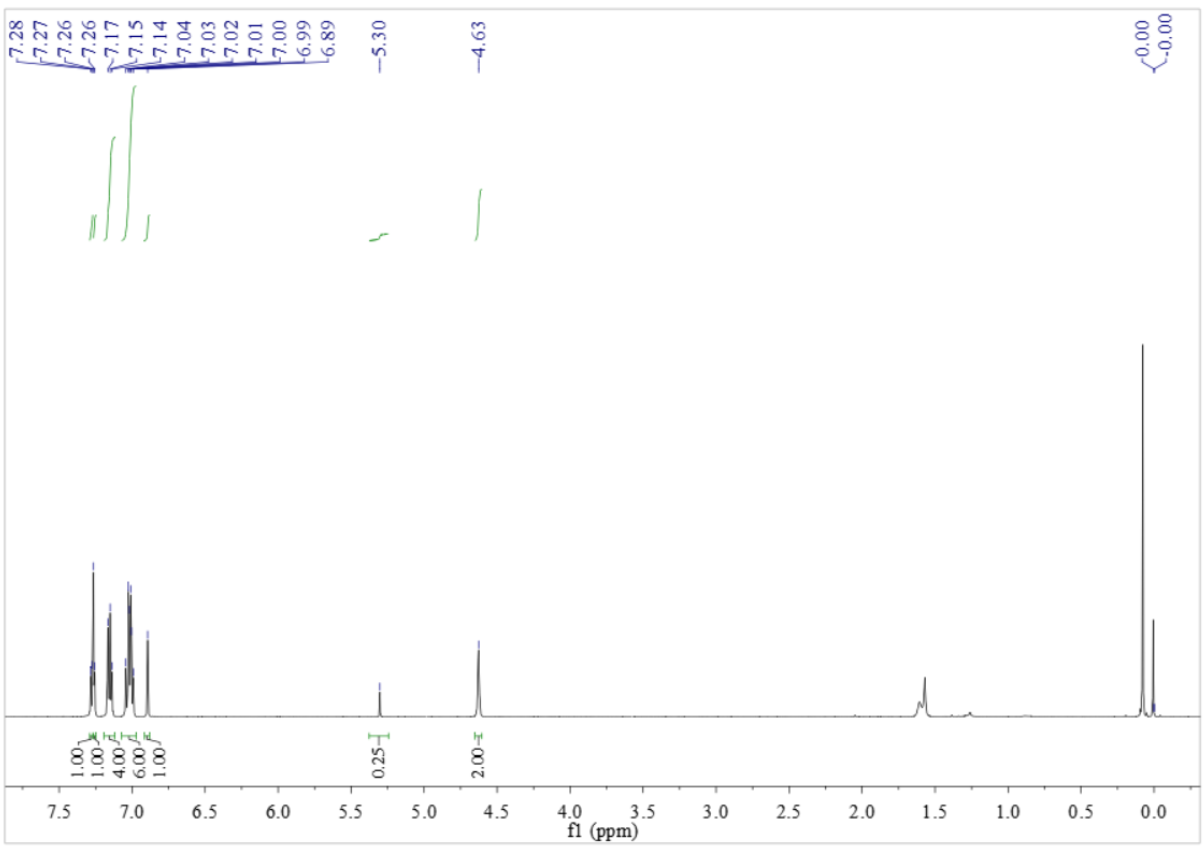


### Figure S16. ^1^H NMR spectrum of TrPEF_2_-A (in Chloroform-*d*).


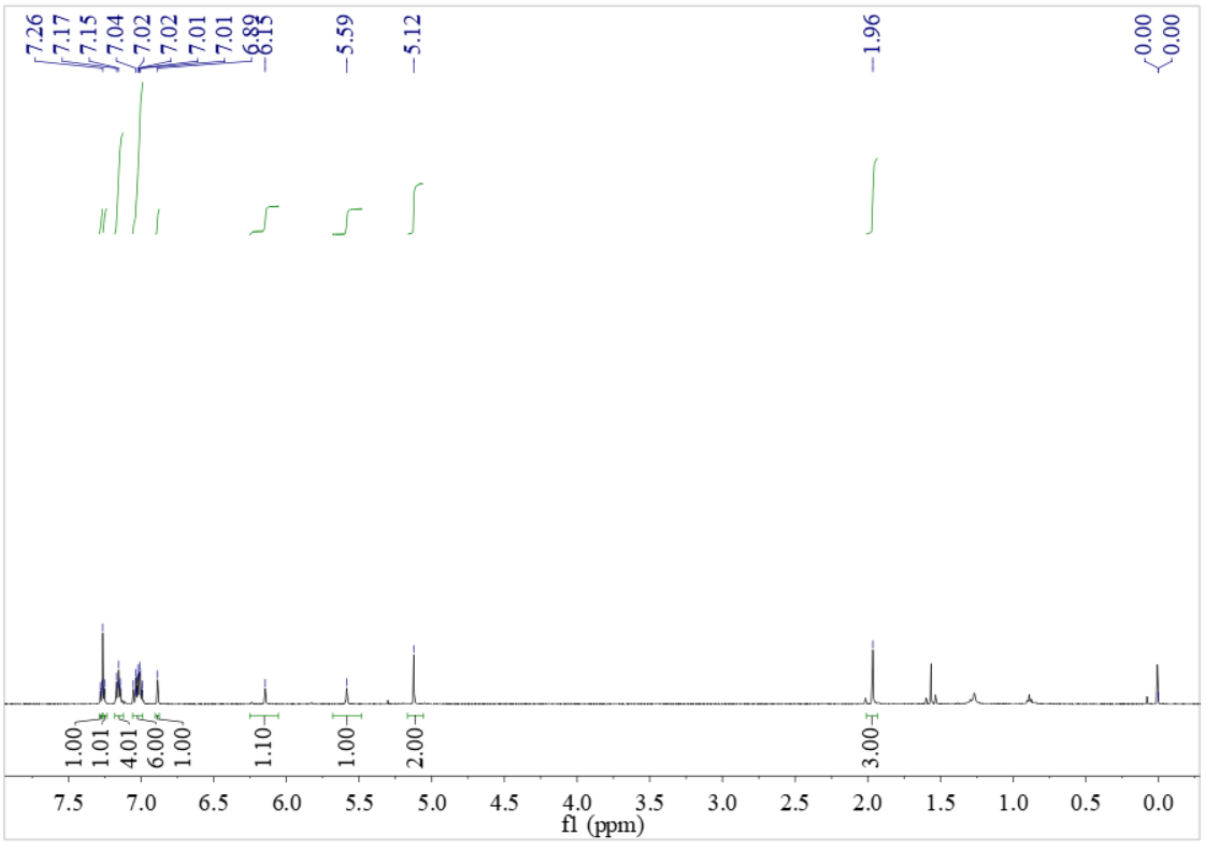


Figure S17. ^1^H NMR spectrum of TrPEF_2_-MA (in Chloroform-*d*).


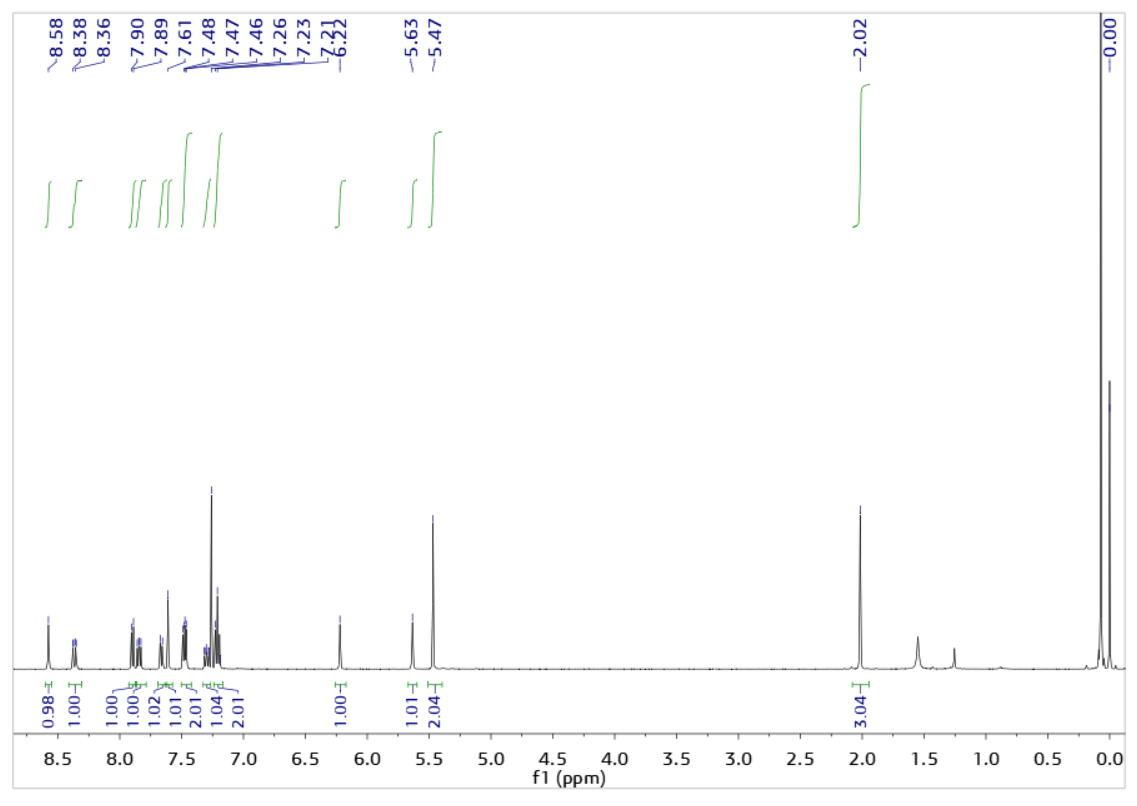


### Figure S18. ^1^H NMR spectrum of TrPEF_2_-MA(O) (in Chloroform-*d*).


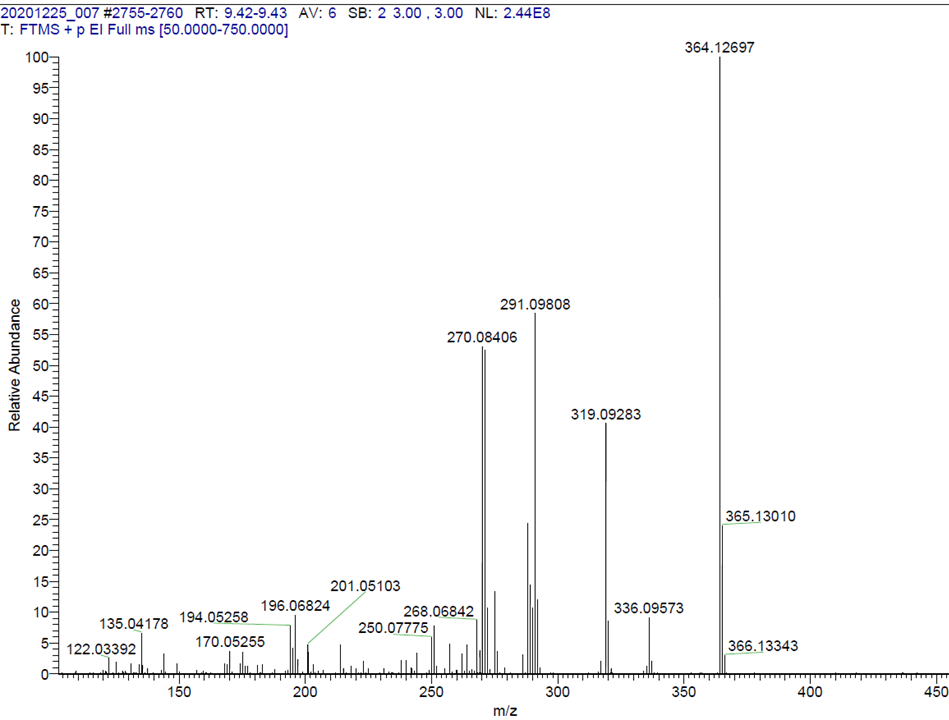


### Figure S19. High resolution Mass spectrum of TrPEF_2_-MF.


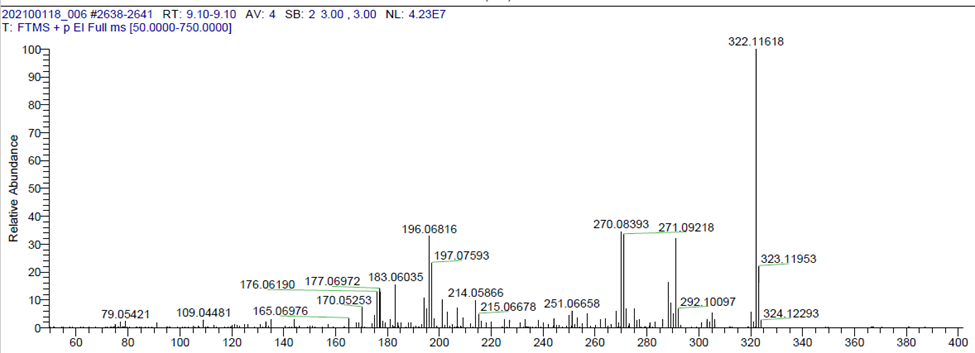


### Figure S20. High resolution Mass spectrum of TrPEF_2_-A.


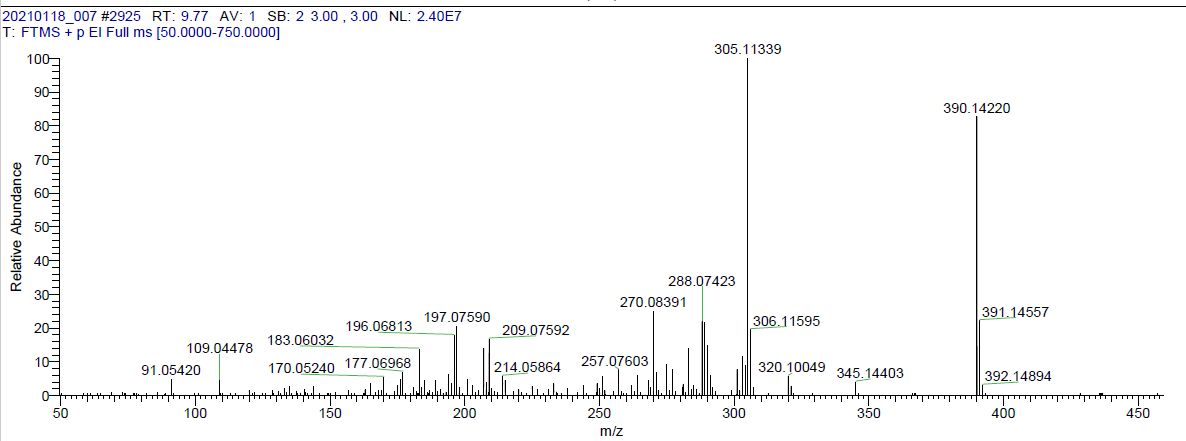


### Figure S21. High resolution Mass spectrum of TrPEF_2_-MA.

Reference

[1] Baker R and Sims RJ. *Synthesis*, 1981, 2, 117.

[2] Ou D, Yu T, Yang Z, et al. *Chemical science*, 2016, 7, 5302-5306.
